# Acute Traumatic Coagulopathy: The Value of Histone in Pediatric Trauma Patients

**DOI:** 10.4274/tjh.2017.0444

**Published:** 2018-05-25

**Authors:** Emel Ulusoy, Murat Duman, Aykut Çağlar, Tuncay Küme, Anıl Er, Fatma Akgül, Hale Çitlenbik, Durgül Yılmaz, Hale Ören

**Affiliations:** 1 Dokuz Eylül University Faculty of Medicine, Department of Pediatric Emergency Care, İzmir, Turkey; 2 Dokuz Eylül University Faculty of Medicine, Department of Biochemistry, İzmir, Turkey; 3 Dokuz Eylül University Faculty of Medicine, Department of Pediatric Hematology, İzmir, Turkey

**Keywords:** Acute traumatic coagulopathy, Children, Histone, Trauma

## Abstract

**Objective:**

Acute traumatic coagulopathy occurs after trauma with impairment of hemostasis and activation of fibrinolysis. Some endogenous substances may play roles in this failure of the coagulation system. Extracellular histone is one such molecule that has recently attracted attention. This study investigated the association between plasma histone-complexed DNA (hcDNA) fragments and coagulation abnormalities in pediatric trauma patients.

**Materials and Methods:**

This prospective case-control study was conducted in pediatric patients with trauma. Fifty trauma patients and 30 healthy controls were enrolled. Demographic data, anatomic injury characteristics, coagulation parameters, computerized tomography findings, trauma, and International Society on Thrombosis and Haemostasis disseminated intravascular coagulation (ISTH DIC) scores were recorded. Blood samples for hcDNA were collected and assessed by enzyme-linked immunosorbent assay.

**Results:**

Thirty-two patients had multiple trauma, while 18 patients had isolated brain injury. hcDNA levels were significantly higher in trauma patients than healthy controls (0.474 AU and 0.145 AU, respectively). There was an association between plasma hcDNA levels and trauma severity. Thirteen patients had acute coagulopathy of trauma shock (ACoTS). ACoTS patients had higher plasma histone levels than those without ACoTS (0.703 AU and 0.398 AU, respectively). Plasma hcDNA levels were positively correlated with the ISTH DIC score and length of stay in the intensive care unit and were negatively correlated with fibrinogen level.

**Conclusion:**

This study indicated that hcDNA levels were increased in pediatric trauma patients and associated with the early phase of coagulopathy. Further studies are needed to clarify the role of hcDNA levels in mortality and disseminated intravascular coagulation.

## Abstract (tr)

**Amaç:**

Akut travma ilişkili koagülopati; travma sonrası ortaya çıkan, hemostazda bozulma ve fibrinoliz aktivasyonudur. Koagülasyon sistemindeki bu bozuklukta bazı endojen moleküller rol oynamaktadır. Histon bu moleküllerden bir tanesi olup son dönemlerde dikkat çekmeye başlamıştır. Bu çalışmada, pediatrik travma olgularında histon-kompleks DNA (hcDNA) fragmanları ile koagülasyon anormallikleri arasındaki ilişkinin incelenmesi amaçlanmıştır.

**Gereç ve Yöntemler:**

Bu çalışma pediatrik travma olgularında yapılmış prospektif olgu-kontrol çalışmasıdır. Çalışmaya 50 hasta ve 30 kontrol olgusu dahil edildi. Tüm hastaların demografik verileri, travmanın özellikleri, koagülasyon parametreleri, bilgisayarlı tomografi sonuçları, travma skorları ve Dissemine İntravasküler Koagülasyon skoru (DİKS) kaydedildi. hcDNA düzeyi için kan örnekleri alınarak enzim ilintili immün test ile değerlendirildi.

**Bulgular:**

Hastaların 32’sinde çoklu travma, 18’inde izole kafa travması mevcuttu. hcDNA düzeyi travma olgularında sağlıklı kontrollere göre istatistiksel olarak anlamlı yüksek bulundu (0,474 AU and 0,145 AU, sırasıyla). Plazma hcDNA düzeyi ile travma ciddiyeti arasında anlamlı ilişki saptandı. On üç hastada akut travma ilişkili koagülopati saptanmış olup, bu hastaların akut travma ilişkili koagülopati olmayanlara göre daha yüksek plazma histon düzeyine sahip oldukları görüldü (0,703 AU and 0,398 AU, sırasıyla). Plazma hcDNA düzeyinin, DİKS ve yoğun bakımda kalış süresi ile pozitif; fibrinojen düzeyi ile negatif korelasyon gösterdiği bulundu.

**Sonuç:**

Bu çalışmada, pediatrik travma olgularında hcDNA düzeyinin arttığı ve koagülopatinin erken fazıyla ilişkili olduğu gösterilmiştir. hcDNA’nın dissemine intravasküler koagülasyon ve mortalite oranını belirlemedeki yerini ortaya koymak için ileri çalışmalara ihtiyaç vardır.

## Introduction

Trauma is the leading cause of visits to pediatric emergency departments [1]. The majority of pediatric trauma is minor, but it remains an important cause of morbidity and mortality in childhood [2]. While massive bleeding is less common in the pediatric trauma cohort, coagulation abnormalities have been described in 10% to 77% of patients [3]. In this regard, identification of coagulopathy and early intervention are important in severely injured trauma patients [4,5].

Acute traumatic coagulopathy (ATC) is an endogenous process that occurs after trauma with the impairment of hemostasis and activation of fibrinolysis [6]. Patients with ATC frequently meet the criteria for disseminated intravascular coagulation (DIC). Currently available data suggest that ATC reflects the early phase of DIC in trauma patients [7,8]. Three major factors that are associated with subsequent development of ATC are hemodilution, hypothermia, and acidosis, and its complex nature is exacerbated by shock and tissue injury [9,10]. Accumulating evidence supports an important role of different interactions between coagulation and inflammation in ATC. Damage-associated molecules such as histone-complexed DNA (hcDNA) fragments released after trauma play a significant role in the balance of the coagulation system [11,12]. There are five types of histones; all have alkaline structures. Histones form an organized pattern with DNA in the cell nucleus by neutralizing the acidic residues of the DNA [13]. Complex structures of DNA, histones, and cell-specific granular proteins, known as neutrophil extracellular traps (NETs), can be released into circulation after stimulation by inflammatory cytokines [14,15]. During NET osis, which is a pathogen-induced cell death causing NET release or tissue damage, nuclear and plasma membranes dissolve or rupture and nuclear materials are released into the circulation [12,16].

DIC can be subdivided into two different phenotypes: fibrinolytic (hemorrhagic) and antifibrinolytic (thrombotic). The antifibrinolytic phenotype is associated with plasminogen activator inhibitor-1 and is seen in sepsis or the late phase of trauma, while the fibrinolytic phenotype leads to coagulopathy including primary and secondary fibrin(ogen)olysis in the early phase of trauma [7]. NETs, contributing factors to coagulopathy in the early stage of trauma, have various effects on the vascular endothelium, platelets, erythrocytes, and coagulation proteins [14,15].Although NETs contain different components like neutrophil granule enzymes and bactericidal molecules, the main structure consists of DNA and histones [16]. hcDNA plays a role in coagulopathy by increasing thrombin formation, activating platelets, stimulating endothelial activation, inhibiting tissue factor pathway inhibitor, causing thrombocytopenia, decreasing fibrinogen, inhibiting anticoagulant protein C activation, and stimulating factor XII-mediated thrombin generation [15,17,18,19,20,21,22,23,24]. In addition, hcDNA complexes, having an integrated linkage between inflammation and coagulation, augment thrombin generation to a greater extent than histones alone [15].

To date, few studies have been done assessing the extracellular hcDNA fragment levels in trauma patients [12,25], while there are no studies investigating this in the pediatric trauma population. The aim of this study was to investigate the relationship of histone with coagulopathy in pediatric trauma patients and also to analyze coagulopathy frequency and its relationship with clinical findings.

## Materials and Methods

### Study Population

This is a prospective case-control study conducted among pediatric patients (1-16 years old) with multiple trauma or isolated brain injury in a pediatric emergency partment between August 2014 and August 2015. Multiple trauma was defined as injury to more than 1 body system, or at least 2 serious injuries to 1 body system [26]. Fifty trauma patients were enrolled in the study. Patients with bleeding diathesis, liver disease, arrival to the trauma center >2 h after injury and/or >40 mL/kg intravenous fluid given before arrival to the hospital, and usage of any drugs including antiplatelet drugs or anticoagulants were all excluded.

Demographic data, patient characteristics, vital signs, and anatomic injury characteristics were recorded.

The control group consisted of 30 children who were evaluated in the outpatient clinic of our hospital for routine well-child visits. None of the children had a history of drug usage, chronic systemic disease, or any major trauma in the last 6 months.

### Scoring Systems

Four scoring systems were used to assess patients upon admission: the Glasgow Coma scale (GCS) [27], the Pediatric Trauma score (PTS) [28], the Injury Severity score (ISS) [29], and the International Society on Thrombosis and Haemostasis (ISTH) DIC score [30]. GCS scores were classified as mild (14-15), moderate (9-13), or severe (3-8) to describe the level of consciousness. The PTS score was determined with six parameters; the minimum score is -6 and the maximum score is +12. Trauma severity is inversely correlated with PTS score and a score of 8 or less indicates the need for trauma services. The ISS consists of six body regions and produces values from 0 to 75. Major trauma is signified by an ISS score of greater than 15. If an injury is assigned an Abbreviated Injury Scale score of 6 (unsurvivable injury), the ISS score is automatically assigned as 75. According to the ISTH DIC scale, overt DIC was diagnosed if the total score was ≥5. The ISTH DIC score includes platelet count, fibrinogen level, prothrombin time (PT), and fibrin degradation products. Age-appropriate reference ranges within our trauma center were used to determine prolonged PT (11.2-14.4 s) and activated partial thromboplastin time (aPTT) (age 1-3 years: 30.6-39.9 s, age 4-7 years: 28.8-38.9 s, age 8-14 years: 28.1-39.1 s, age 14-18 years: 26.0-36.6 s) levels. The presence of acute coagulopathy of trauma shock (ACoTS) was defined as prolonged PT and/or aPTT according to the age-appropriate references ranges [31,32,33,34,35].

The threshold for defining anemia is hemoglobin at or below the 2.5^th ^percentile for age, race, and sex [36]. Hypothermia is graded as mild (36-34 °C), moderate (34-32 °C), or severe (<32 °C) [37].

### Biochemical Analysis

Biochemical parameters of the study population were assessed on admission. Venous blood gas, routine biochemistry, complete blood count, PT, aPTT, fibrinogen, and D-dimer were evaluated based on normal laboratory reference ranges in the hospital.

Peripheral venous blood samples were collected in blood tubes with EDTA for hcDNA. Tubes were centrifuged at 1200 x g for 10 min and plasma samples were stored at -80 °C until analysis. Plasma nucleosome levels were measured with the Cell Death Detection ELISA^PLUS^ commercial kit based on the principle of sandwich enzyme immunoassay (Catalog No: 1774425, Roche Diagnostics, Mannheim, Germany). Results were reported as absorbance units (AU).

### Ethical Approval

The study protocol was designed in compliance with the Declaration of Helsinki. Informed consent was obtained from parents or legal guardians before enrollment in the study. The study was begun after receiving the approval of the Ethics Committee of the Dokuz Eylül University Faculty of Medicine.

### Statistical Analysis

Statistical analysis was performed using SPSS 22.0 (IBM Corp., Armonk, NY, USA). Power hoc analysis was performed to evaluate the sample size. Data are presented as medians with interquartile ranges (IQRs) and 25^th^-75^th^ percentiles. Histograms were used to assess the normality of sample distributions. The Kruskal-Wallis test was used for analyzing plasma hcDNA levels among different groups. The Mann-Whitney U test was used for comparing two groups. The chi-square test was used for comparing group ratios. Correlations between parameters were computed through Pearson correlation analysis. All t-tests were two-tailed and group differences or correlations with p<0.05 were considered to be statistically significant. Receiver operating curve (ROC) analysis was used to detect the optimal cut-off points for separating the ACoTS group from the healthy control group. Bonferroni correction was used for multiple comparisons.

## Results

Fifty trauma patients and 30 healthy controls were enrolled in the study. The study group and control children were comparable in terms of age and sex distribution (Table 1).

Falls were the most frequent cause of injury; the second most common was motor vehicle accidents (Table 2).

Eighteen (36%) patients had isolated brain injury while 32 (64%) patients had multiple trauma. Three patients had liver laceration, 3 patients had spleen laceration, and 1 patient had renal and spleen laceration. Twenty-one patients had anemia and none of the patients had thrombocytopenia. Although no patient had overt DIC, 13 patients had ACoTS. Ten patients had only prolonged PT, 1 patient had only prolonged aPTT, and 2 patients had both. The median level of PT was 13.0 s (12.3-14.2 s) and the median level of aPTT was 27.6 s (23.7-30.4 s) in trauma patients. There were significant differences between patients with ACoTS [PT: 15.6 s (14.9-16.9 s), aPTT: 31.8 s (27.6-40.0 s)] and those without ACoTS [PT: 12.6 s (12.1-13.4 s), aPTT: 25.9 s (22.3-29.1 s)] according to hemostasis parameters (p=0.000 and p=0.001, respectively). Nineteen patients (38%) were admitted to the intensive care unit (ICU). Emergency endotracheal intubation was performed for 15 patients. The overall mortality rate was 6%. Clinical characteristics of the patients are shown in Table 3.

When we evaluated patients according to the ISS, we determined that 21 patients had scores over 16 and only three patients had 75 points. Those three patients died.

**Figure 1:**
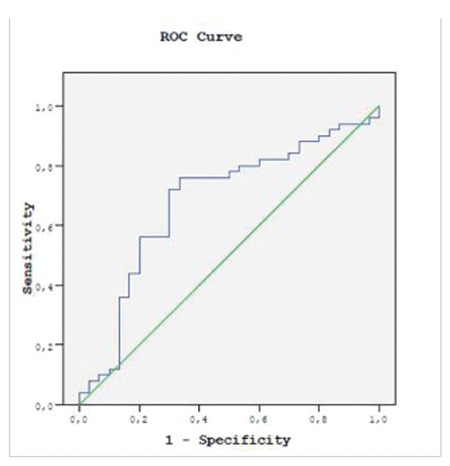
Receiver operating characteristic curve analyses of histone-complexed DNA.

Plasma hcDNA levels were significantly higher in trauma patients [0.474 AU (0.184-0.841 AU)] than in healthy controls [0.145 AU (0.086-0.361 AU)] (p=0.008). ACoTS patients [0.703 AU (0.301-0.897 AU)] had higher plasma histone levels than those without ACoTS [0.398 AU (0.130-0.802 AU)]. We found significant differences between hcDNA levels and groups according to the GCS, PTS, ISS, and D-dimer (Table 4), but we did not find any differences of hcDNA levels in terms of trauma type (p=0.338). ROC curve analyses of hcDNA were performed. ROC analysis revealed an optimal cut-off point at 0.186 AU for separating the ACoTS patients from the control group. The sensitivity and specificity were 76.0% and 66.4%, respectively. The area under the curve for hcDNA was 0.679 (p=0.008) (Figure 1).

Plasma hcDNA levels were significantly correlated with ISTH DIC score (r=0.433, p=0.002) and length of stay in the ICU (r=0.314, p=0.026) in the whole study group. There was a negative correlation between hcDNA levels and PTS score (r=-0.464, p=0.001). When we investigated coagulation parameters, we found a positive correlation between hcDNA levels and D-dimer levels (r=0.597, p≤0.001) and a negative correlation with fibrinogen (r=-0.342, p=0.015).

While most of the patients with organ laceration had higher hcDNA levels than the median level in the trauma group, we did not find any differences in hcDNA levels according to pathological findings in computerized tomography (CT) of the brain, thorax, or abdomen (p=0.342, p=0.229, and p=0.071, respectively).

## Discussion

In the present study, blood levels of hcDNA fragments and ATC were assessed in pediatric trauma patients. The findings showed that extracellular histone correlates with ATC and also trauma severity.

In this study, ACoTS was determined in 13 (26%) patients. In the literature there is a wide range, varying from 10% to 71%, for the incidence of coagulopathy on admission after severe trauma in the pediatric population [3]. Routine coagulation tests such as PT, aPTT, international normalized ratio, fibrinogen, or fibrinogen degradation product have been used to determine the presence of coagulopathy, but Mann et al. [38] showed that these tests do not indicate whole coagulation system abnormalities because of reflecting only 4% of thrombin production. Different studies have been performed to clarify mechanisms of coagulopathy after trauma. In a large retrospective study it was found that large-volume resuscitation with fluid during the management of shock causes dilution of plasma proteins and coagulation factors [39], but Brohi et al. [35] also showed that coagulopathy could occur before excessive fluid resuscitation. Acidosis and hypothermia can be easily observed in patients with especially severe trauma, which cause clotting and platelet dysfunction. Dirkmann et al. [40] showed that if both of them existed, a synergistic effect occurred on coagulopathy and mortality was increased. These factors increase the coagulopathy risks after trauma, but the exact nature of this process is still not clear and correction of acidosis and hypothermia does not always correct the associated coagulopathy. This has led researchers to continue investigating the additional underlying mechanisms. Damage-associated molecules play a significant role in the balance of the coagulation system in critically ill children [10,14]. In the current study, we demonstrated the increase of hcDNA in the early phase of coagulopathy without existing DIC in pediatric trauma patients.

This study showed that plasma hcDNA levels were higher in the trauma group than in healthy controls. This increase occurs because of nuclear proteins being released out of the cell membrane with cells dying in critically ill patients and in cases of trauma [41]. As is well known, nuclear and plasma membranes must be damaged for the release of intranuclear substances like histone or DNA to occur after mechanical trauma [42]. In this regard, extracellular hcDNA levels must be higher as trauma becomes more serious with growing tissue damage. Kutcher et al. [25] showed that critically injured adult trauma patients with high hcDNA levels had higher ISS and lower GCS scores. In another study of adults, Johansson et al. [12] found a correlation between the circulating hcDNA levels and ISS values. In accordance with the literature, this study shows a relationship between plasma hcDNA levels and trauma severity according to GCS, PTS, and ISS scores. According to the GCS, the highest hcDNA level was seen in the moderate GCS group. Multiple organ injuries were mainly found in the moderate GCS group. Among these trauma patients, only 2 patients had serious organ injuries other than head trauma in the severe GCS group. The amount of tissue damage was highest in the moderate GCS group and lowest in the mild GCS group, consistent with histone levels.

The main finding of our study was that plasma hcDNA levels were significantly correlated with coagulation parameters that indicate coagulopathy in the pediatric trauma population. After tissue injury, histone moves out of the cell membrane and increases activated protein C (aPC). In turn, aPC inhibits FV, FVIII, and PAI-1, thereby creating hypocoagulation and hyperfibrinolysis [43]. The late phase of trauma can be complicated with hypercoagulability and thromboembolic events like prothrombotic states after depletion of aPC stores as reported in septic patients [44]. In addition, extracellular histone activates platelets by TLR2 and TLR4 to cause platelet aggregation [17]. In experimental models with mice, histone injection caused coagulopathy and bleeding with prolonged PT, decreased fibrinogen, and fibrin deposition [45]. In this respect, it seems that histone plays roles in both pro- and anticoagulant processes. Two human studies examined blood histone levels and coagulopathy in adult trauma patients [12,25]. The present study demonstrated a hypocoagulopathic phase at the early stage of trauma having an association between histone levels and increased PT and aPTT and decreased fibrinogen.

Another result presented here is plasma hcDNA levels being correlated with length of stay in the ICU in the whole study group. A relationship between elevated histone levels and days of mechanical ventilation was found in trauma patients by Kutcher et al. [25]. This result is not surprising considering that patients with high histone levels had high trauma severity and coagulopathy. The effects of histone on lung tissue were also shown in human and animal models. High histone levels caused 1.8-fold higher incidence of acute lung injury [25] along with pulmonary edema, hemorrhage, and microvascular thrombosis after injection of histone in animal models [46]. Nakahara et al. [45] showed that extracellular histones caused platelet aggregation, thrombotic occlusion of pulmonary capillaries, and right-sided heart failure. We could not show a relationship between histone levels and thorax, cranial, or abdominal CT findings. Because these patients had multiple trauma, we could not isolate any organ systems from the other tissues. When these patients had higher ISS values, we attributed it to the release of histone from dying cells that could not be shown by imaging methods. We are unable to discuss whether a high histone level is a marker for mortality due to the small size of our patient population.

Treatment approaches have undergone more investigation since the understanding of the important role of NETs in coagulopathy. NETs may represent an attractive target for antithrombotic therapy. In the literature, prevention of histone toxicity on platelets and protection against histone-induced thrombocytopenia by heparin were demonstrated [17,18]. Nakahara et al. [45] also demonstrated that recombinant thrombomodulin protects mice against histone-induced lethal thromboembolism. New therapeutic approaches have caused excitement, with the better understanding of NETs including hcDNA contributing to the pathophysiology of coagulopathy.

## Conclusion

In conclusion, this study indicated that hcDNA levels increase in pediatric trauma patients associated with coagulopathy. There was an association between plasma hcDNA levels and trauma severity according to GCS, PTS, and ISS scores. There was also a significant correlation between hcDNA levels and length of stay in the ICU. Further studies are needed to clarify the role of high hcDNA levels in determining the functional significance of these changes in therapy, DIC, and prediction of mortality.

## Figures and Tables

**Table 1:**
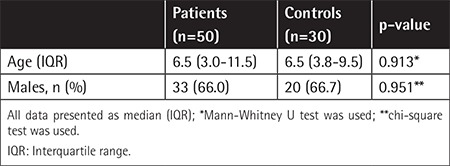
Demographic variables of patients and controls.

**Table 2:**
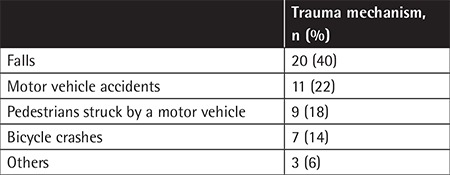
Trauma mechanisms in the whole patient group.

**Table 3:**
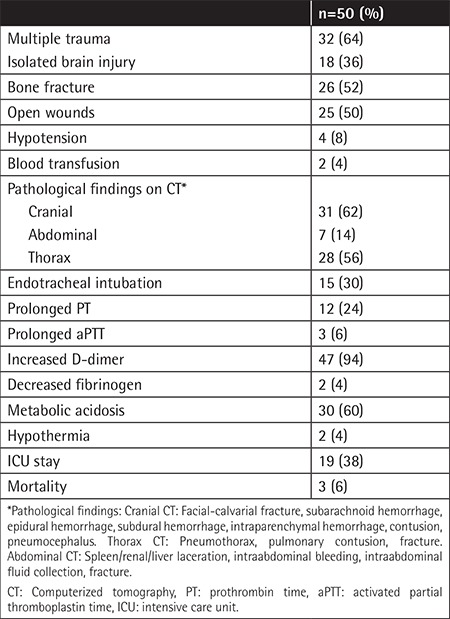
Clinical and laboratory characteristics of the patients.

**Table 4:**
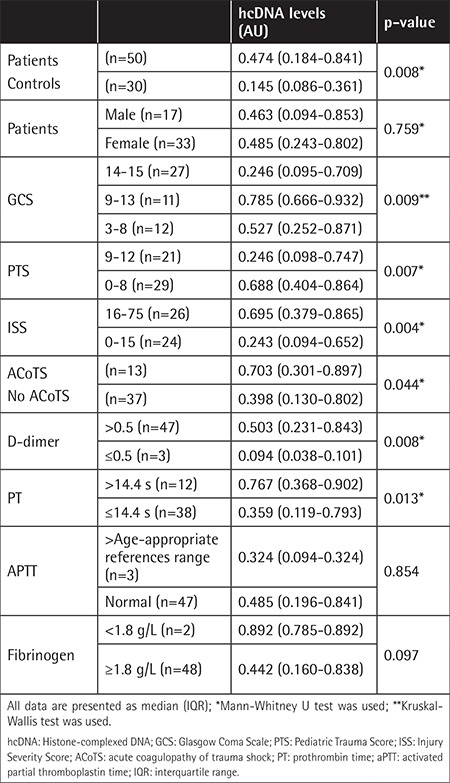
Histone-complexed DNA levels of patients and controls.

## References

[1] Wier LM, Hao Y, Owens P, Washington R (2013.). Overview of Children in the Emergency Department, 2010. CUP Statistical Brief #157. Rockville, Agency for Healthcare Research and Quality.

[2] Avarello JT, Cantor RM (2007). Pediatric major trauma: an approach to evaluation and management. Emerg Med Clin North Am.

[3] Christians SC, Duhachek-Stapelman AL, Russell RT, Lisco SJ, Kerby JD, Pittet JF (2014). Coagulopathy after severe pediatric trauma. Shock.

[4] Rourke C, Curry N, Khan S, Taylor R, Raza I, Davenport R, Stanworth S, Brohi K (2012). Fibrinogen levels during trauma hemorrhage, response to replacement therapy, and association with patient outcomes. J Thromb Haemost.

[5] Epstein DS, Mitra B, Cameron PA, Filtzgerald M, Rosenfeld JV (2016). Normalization of coagulopathy is associated with improved outcome after isolated traumatic brain injury. J Clin Neurosci.

[6] Brohi K, Cohen MJ, Ganter MT, Schultz MJ, Levi M, Mackersie RC, Pittet JF (2008). Acute coagulopathy of trauma: hypoperfusion induces systemic anticoagulation and hyperfibrinolysis. J Trauma.

[7] Gando S, Otomo Y (2015). Local hemostasis, immunothrombosis, and systemic disseminated intravascular coagulation in trauma and traumatic shock. Crit Care.

[8] Gando S (2009). Acute coagulopathy of trauma shock and coagulopathy of trauma: a rebuttal. You are now going down the wrong path. J Trauma.

[9] Frith D, Goslings JC, Gaarder C, Maegele M, Cohen MJ, Allard S, Johansson PI, Stanworth S, Thiemermann C, Brohi K (2010). Definition and drivers of acute traumatic coagulopathy: clinical and experimental investigations. J Thromb Haemostasis.

[10] Davenport R (2013). Pathogenesis of acute traumatic coagulopathy. Transfusion.

[11] Zhang Q, Raoof M, Chen Y, Sumi Y, Sursal T, Junger W, Brohi K, Itagaki K, Hauser CJ (2010). Circulating mitochondrial DAMPs cause inflammatory responses to injury. Nature.

[12] Johansson PI, Windelov NA, Rasmussen LS, Sørensen AM, Ostrowski SR (2013). Blood levels of histone-complexed DNA fragments are associated with coagulopathy, inflammation and endothelial damage early after trauma. J Emerg Trauma Shock.

[13] Felsenfeld G, Groudine M (2003). Controlling the double helix. Nature.

[14] Kim JE, Lee N, Gu JY, Yoo HJ, Kim HK (2015). Circulating levels of DNA histone complex and dsDNA are independent prognostic factors of disseminated intravascular coagulation. Thromb Res.

[15] Wisher JW, Becker RC (2014). Antithrombotic therapy: new areas to understand efficacy and bleeding. Expert Opin Ther Targets.

[16] Gould TJ, Lysov Z, Liaw PC (2015). Extracellular DNA and histones: double-edged swords in immunothrombosis. J Thromb Haemost.

[17] Semeraro F, Ammollo CT, Morrissey JH, Dale GL, Friese P, Esmon NL, Esmon CT (2011). Extracellular histones promote thrombin generation through platelet-dependent mechanisms: involvement of platelet TLR2 and TLR4. Blood.

[18] Fuchs TA, Bhandari AA, Wagner DD (2011). Histones induce rapid and profound thrombocytopenia in mice. Blood.

[19] Martinod K, Wagner DD (2014). Thrombosis: tangled up in NETs. Blood.

[20] Ammollo CT, Semeraro F, Xu J, Esmon NL, Esmon CT (2011). Extracellular histones increase plasma thrombin generation by impairing thrombomodulin-dependent protein C activation. J Thromb Haemost.

[21] Komissarov AA, Florova G, Idell S (2011). Effects of extracellular DNA on plasminogen activation and fibrinolysis. J Biol Chem.

[22] Barranco-Medina S, Pozzi N, Vogt AD, Di Cera E (2013). Histone H4 promotes prothrombin autoactivation. J Biol Chem.

[23] Xu J, Zhang X, Pelayo R, Monestier M, Ammollo CT, Semeraro F, Taylor FB, Esmon NL, Lupu F, Esmon CT (2009). Extracellular histones are major mediators of death in sepsis. Nat Med.

[24] Altincicek B, Stotzel S, Wygrecka M, Preissner KT, Vilcinskas A (2008). Host-derived extracellular nucleic acids enhance innate immune responses, induce coagulation, and prolong survival upon infection in insects. J Immunol.

[25] Kutcher ME, Xu J, Vilardi RF, Ho C, Esmon CT, Cohen MJ (2012). Extracellular histone release in response to traumatic injury: implications for a compensatory role of activated protein C. J Trauma Acute Care Surg.

[26] Letts M, Davidson D, Lapner P (2002). Multiple trauma in children: predicting outcome and long-term results. Can J Surg.

[27] Teasdale G, Jennett B (1974). Assessment of coma and impaired consciousness. A practical scale. Lancet.

[28] Furnival RA, Schunk JE (1999). ABCs of scoring systems for pediatric trauma. Pediatr Emerg Care.

[29] ((accessed on 21 August 2013).). Trauma.org. Injury Severity Score.

[30] Taylor FB Jr, Toh CH, Hoots WK, Wada H, Levi M, Scientific Subcommittee on Disseminated Intravascular Coagulation (DIC) of the International Society on Thrombosis and Haemostasis (ISTH) (2001). Towards definition, clinical and laboratory criteria, and a scoring system for disseminated intravascular coagulation on behalf of the Scientific Subcommittee on Disseminated Intravascular Coagulation of the International Society on Thrombosis and Haemostasis. Thromb Haemost.

[31] Macleod JB, Lynn M, McKenney MG, Cohn SM, Murtha M (2003). Early coagulopathy predicts mortality in trauma. J Trauma.

[32] Johansson PI, Sørensen AM, Perner A, Welling KL, Wanscher M, Larsen CF, Ostrowski SR (2011). Disseminated intravascular coagulation or acute coagulopathy of trauma shock early after trauma? An observational study. Crit Care.

[33] Johansson PI, Stensballe J, Rasmussen LS, Ostrowski SR (2012). High circulating adrenaline levels at admission predict increased mortality after trauma. J Trauma Acute Care Surg.

[34] Curry NS, Davenport RA, Hunt BJ, Stanworth SJ (2012). Transfusion strategies for traumatic coagulopathy. Blood Rev.

[35] Brohi K, Singh J, Heron M, Coats T (2003). Acute traumatic coagulopathy. J Trauma.

[36] Brugnara C, Oski FA, Nathan DG, Orkin SH, Nathan DG, Ginsburg D, Look AT, Fisher DE, Lux S (2015). Diagnostic approach to the anemic patient. Nathan and Oski’s Hematology and Oncology of Infancy and Childhood, 8th ed.

[37] Tsuei BJ, Kearney PA (2004). Hypothermia in the trauma patient. Injury.

[38] Mann KG, Butenas S, Brummel K (2003). The dynamics of thrombin formation. Arterioscler Thromb Vasc Biol.

[39] Maegele M, Lefering R, Yucel N, Tjardes T, Rixen D, Paffrath T, Simanski C, Neugebauer E, Bouillon B, AG Polytrauma of the German Trauma Society (DGU) (2007). Early coagulopathy in multiple injury: an analysis from the German Trauma Registry on 8724 patients. Injury.

[40] Dirkmann D, Hanke AA, Görlinger K, Peters J (2008). Hypothermia and acidosis synergistically impair coagulation in human whole blood. Anesth Analg.

[41] Allam R, Kumar SV, Darisipudi MN, Anders HJ (2014). Extracellular histones in tissue injury and inflammation. J Mol Med (Berl).

[42] Hotchkiss RS, Strasser A, McDunn JE, Swanson PE (2009). Cell death. N Engl J Med.

[43] Fulcher CA, Gardiner JE, Griffin JH, Zimmerman TS (1984). Proteolytic inactivation of human factor VIII procoagulant protein by activated human protein C and its analogy with factor V. Blood.

[44] Esmon CT (2002). Protein C pathway in sepsis. Ann Med.

[45] Nakahara M, Ito T, Kawahara K, Yamamoto M, Nagasato T, Shrestha B, Yamada S, Miyauchi T, Higuchi K, Takenaka T, Yasuda T, Matsunaga A, Kakihana Y, Hashiguchi T, Kanmura Y, Maruyama I (2013). Recombinant thrombomodulin protects mice against histone-induced lethal thromboembolism. PLoS One.

[46] Abrams ST, Zhang N, Manson J, Liu T, Dart C, Baluwa F, Wang SS, Brohi K, Kipar A, Yu W, Wang G, Toh CH (2013). Circulating histones are mediators of trauma associated lung injury. Am J Respir Crit Care Med.

